# Two-time perforation of the ileal J-pouch 6 and 18 years after restorative proctocolectomy and ileal pouch–anal anastomosis for familial adenomatous polyposis: a case report

**DOI:** 10.1186/s40792-021-01355-9

**Published:** 2022-01-04

**Authors:** Kengo Shibata, Shota Ebinuma, Sodai Sakamoto, Asami Suzuki, Yasunobu Terasaki, Akinobu Taketomi

**Affiliations:** 1 Division of Surgery, Wakkanai City Hospital, 03-13-15, Chuo, Wakkanai City, Hokkaido 097-8686 Japan; 2 Department of Gastroenterological Surgery I, Hokkaido University Graduate School of Medicine, Kita 15, Nishi 7, Kita-ku, Sapporo, Hokkaido 060-8638 Japan; 3 Division of Nursing, Wakkanai City Hospital, 03-13-15, Chuo, Wakkanai City, Hokkaido 097-8686 Japan

**Keywords:** Familial adenomatous polyposis, Ileal pouch–anal anastomosis, Restorative proctocolectomy, Perforation

## Abstract

**Background:**

Perforation of the ileal J-pouch after restorative proctocolectomy and ileal pouch–anal anastomosis are extremely rare. There has been no report of perforation of the ileal J-pouch occurring twice over several years. We report the first case of perforation at 6 and 18 years following restorative proctocolectomy.

**Case presentation:**

The patient was a 52-year-old man who underwent a two-stage restorative proctocolectomy with a hand-sewn ileal J-pouch anal anastomosis due to familial adenomatous polyposis and sigmoid colon cancer at 34 years of age. At the age of 40, he underwent ileal pouch resection at its blind end, abdominal drainage, and anastomotic dilatation. The patient had a perforation of the blind end of the ileal J-pouch from increased intraluminal pressure, with anastomotic stricture and pervasive peritonitis. The patient had no symptoms for a few years; however, 18 years after the initial surgery and 12 years after the first perforation, the patient presented with severe abdominal pain. Computed tomography demonstrated pneumoperitoneum; accordingly, laparotomy was performed. Upon opening the abdominal cavity, contaminated ascites and inflammatory changes were documented involving the ileum. A 2-mm perforation involving the blind end of the ileal J-pouch was also observed and repaired, followed by temporary loop ileostomy creation. Postoperative endoscopy revealed an ulcer in the ileal J-pouch and a stricture located directly at the anastomosis.

**Conclusions:**

The blind end of the J-pouch repeatedly perforated over the years due to recurrent anastomotic stricture. Regular surveillance is, therefore, considered necessary for the release of stricture, maintenance of anastomotic patency, and prevention of ileal J-pouch perforation.

## Background

Familial adenomatous polyposis (FAP) is a genetic complex syndrome estimated to affect 1–8 out of every 1000 people. It has a high incidence of more than 100 adenomas in the colon, and almost 100% of patients with this condition develop colorectal cancers by the age of 60 years [1, 2]. Therefore, prophylactic restorative proctocolectomy (RPC) is often performed, and currently, RPC and ileal pouch–anal anastomosis (IPAA) are considered the standard techniques [3, 4]. Complications of IPAA include pouchitis (18.8%), pelvic sepsis (9.5%), and other conditions [5]. However, perforation of the ileal pouch itself is extremely rare, and there have been no reports of a second perforation occurring over the years. We report the first case of ileal pouch perforation that occurred 6 and 18 years after total colorectal resection.

## Case presentation

**Fig. 1:**
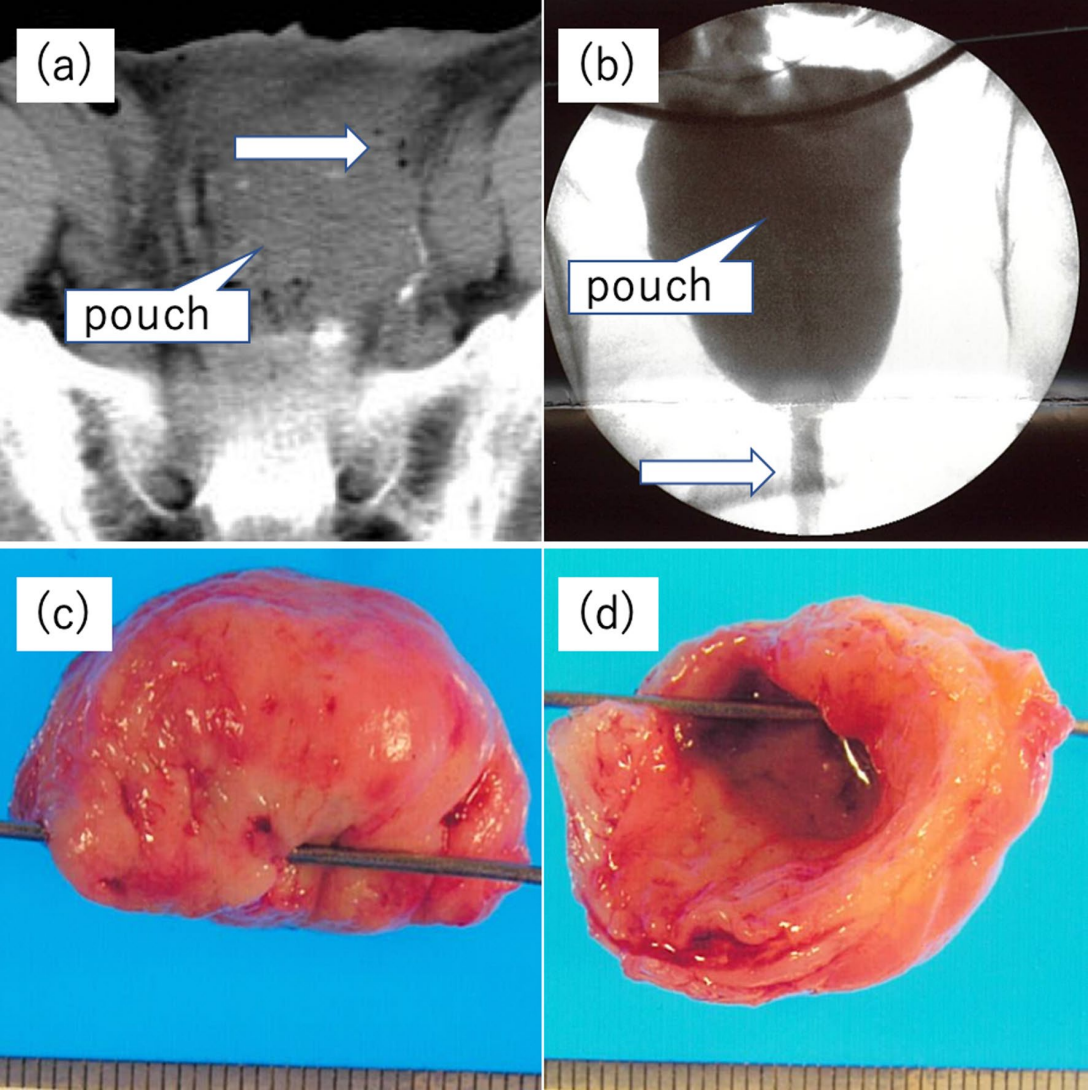
Findings upon initial perforation. **a** Computed tomography. CT revealed free air and fluid collection around the blind end of the J-pouch (arrow), and perforation of the digestive tract was suspected. **b** Gastrointestinal gastrographic findings during surgery. Gastrointestinal gastrography revealed an anastomotic stricture. This appeared to cause an increase in the intraluminal pressure in the J-pouch. **c**, **d** Excised specimen findings. Gross appearance of the excised specimen. A 2-mm perforation was observed at the top of the blind end of the J-pouch. Inflammatory findings were observed on the serous surface (**c**). No ulcers were observed on the mucosal surface (**d**)

**Fig. 2:**
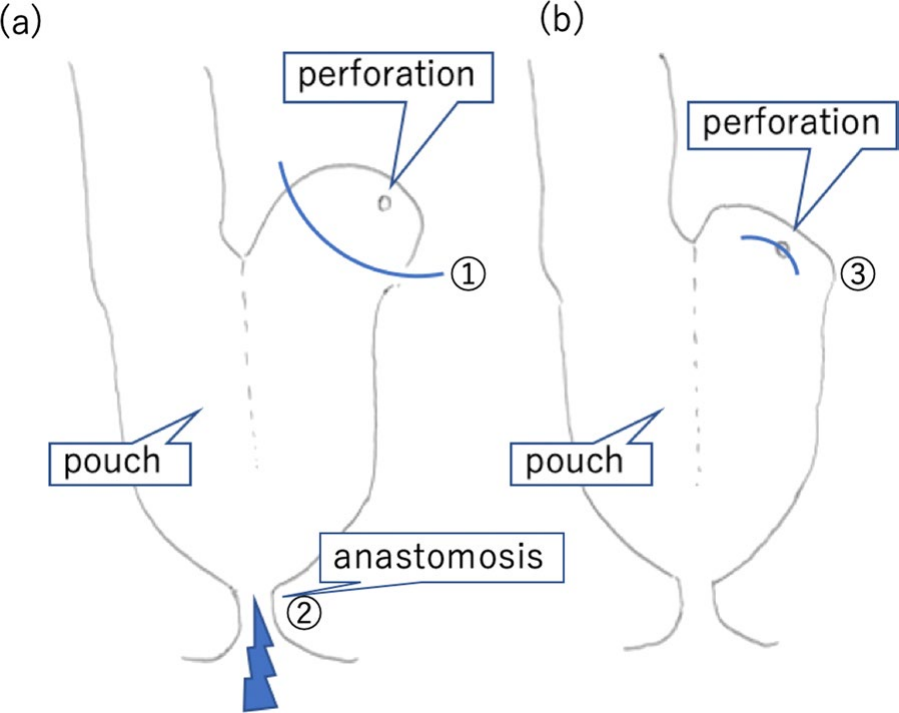
Schemes of the operation. **a** Operation during the first perforation. A 2-mm perforation at the top of the pouch was resected using a stapler ①. An anastomotic stricture was observed, and it was manually dilated to 20 mm in diameter ②. **b** Operation during the second perforation. A 2-mm perforation at the top of the pouch was once again observed and primary repair was performed ③. Temporary loop ileostomy was performed

**Fig. 3:**
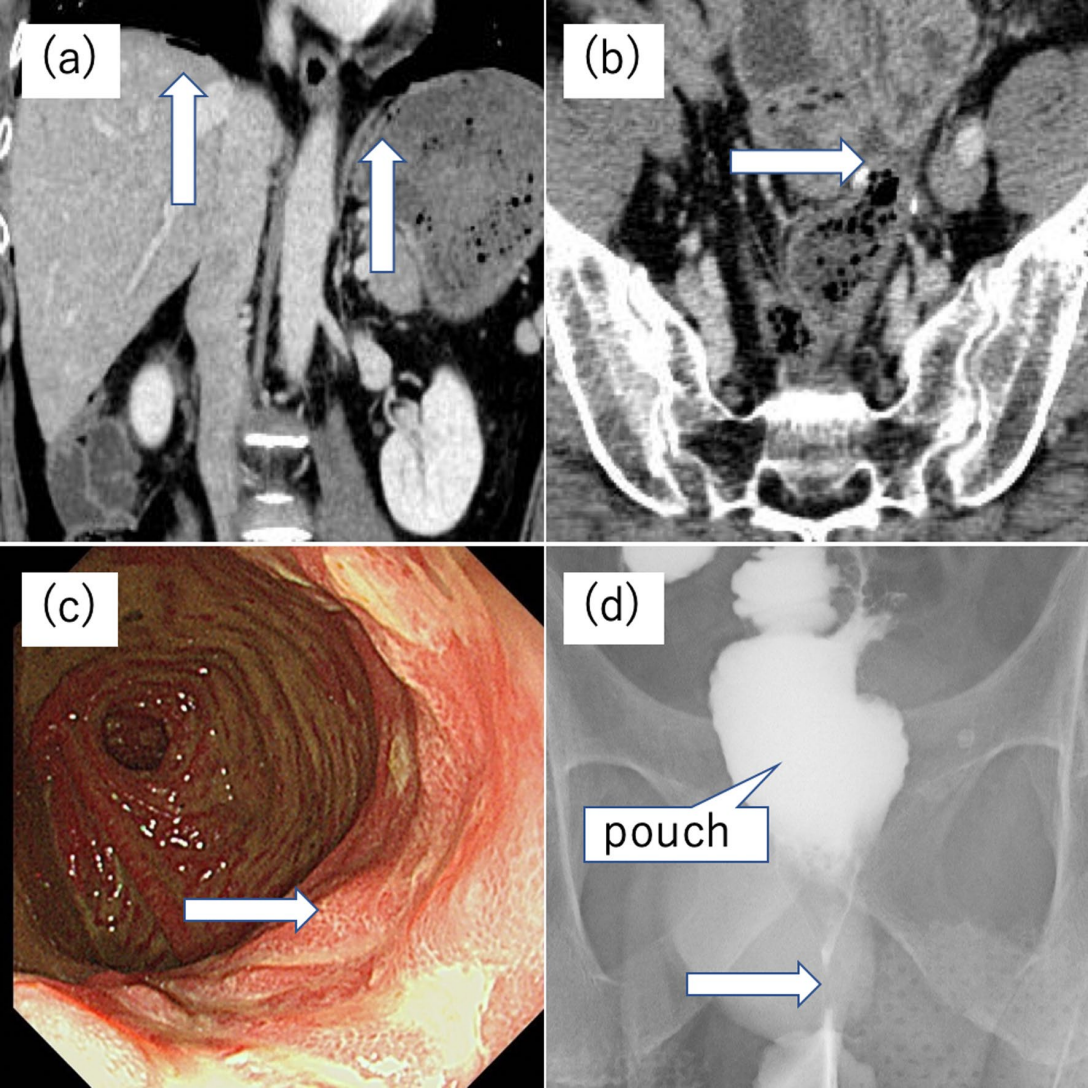
Findings upon the second perforation. **a**, **b** Contrast-enhanced computed tomography. Contrast-enhanced CT revealed free air (arrow: **a**) and fluid collection around the blind end of the J-pouch (arrow: **b**). **c** Endoscopic observation and gastrointestinal gastrography findings after surgery. Endoscopy revealed multiple ulcers in the ileal J-pouch, and gastrointestinal gastrography revealed an anastomotic stricture. **d** Gastrointestinal gastrographic findings after surgery. Gastrointestinal gastrography revealed an anastomotic stricture again

A 52-year-old man presented with FAP. At the age of 34 years, he underwent a two-stage restorative proctocolectomy and a hand-sewn ileal J-pouch anal anastomosis at a previous hospital because of FAP co-presenting with sigmoid colon cancer. The clinical stage according to the TNM 8th edition was T3 N1b M0, Stage IIIB. Adjuvant chemotherapy was not prescribed and regular observation was undertaken. He had no other symptoms, including constipation, and he did not visit the hospital for follow-up 1 year postoperatively. However, 6 years following surgery, he experienced abdominal distension and constipation. He visited our emergency department with complaints of severe pain in his lower abdomen. On physical examination, signs of peritoneal irritation were observed throughout the abdomen, and anal strictures were palpable upon digital examination. Laboratory examination demonstrated an elevated white blood cell (WBC) count of 13,800/μl and a C-reactive protein (CRP) level of 2.19 mg/dl. Computed tomography (CT) of the abdomen revealed a pneumoperitoneum (Fig. 1a). Perforation of the digestive tract was also diagnosed. Accordingly, laparotomy was performed on the day of admission. A large amount of cloudy ascites in the abdominal cavity was observed, and the blind end of the ileal J-pouch was enlarged. A 2-mm perforation was observed at the top of the pouch, and the upper part of the blind end was resected using a stapler (Fig. 2a-①). Gastrointestinal gastrography during surgery revealed an anastomotic stricture (Fig. 1b). The perforation was found to have been caused by increased intraluminal pressure in the extended pouch from the anastomotic stricture; hence, the anastomotic stricture was manually dilated to 20 mm in diameter (Fig. 2a-②). Histopathological examination revealed no significant findings on the mucosal or muscular surfaces, and only inflammatory findings on the serous surface were observed (perforation and peritonitis) (Fig. 1c, d). The patient was discharged 22 days postoperatively. Following this episode, the patient had no symptoms and was unable to visit the hospital for follow-up. Eighteen years after initial surgery (12 years after the first perforation), he presented with severe abdominal pain. Laboratory examinations revealed an elevated WBC count of 17,900/μl and CRP level of 1.79 mg/dl. CT demonstrated free air in the abdomen (Fig. 3a, b), and physical examination revealed that pain and the abdominal signs, which were mild at the time of presentation, gradually increased in severity until peritoneal irritation symptoms were subsequently observed throughout. Therefore, intestinal perforation was suspected, and laparotomy was performed. Contaminated ascites and inflammation of the ileum in the abdominal cavity and a 2-mm perforation at the top of the ileal J-pouch were once again observed. The perforation was repaired followed by abdominal intraperitoneal drainage and temporary loop ileostomy (Fig. 2a-③). Postoperative endoscopy revealed multiple mucosal ulcers (Fig. 3c), and gastrointestinal gastrography revealed stricture of the anastomotic segment (Fig. 3d). The patient was discharged 27 days after surgery without complications. After 1 month, the multiple mucosal ulcers improved without antibiotics.

## Discussion

FAP is an autosomal dominant inherited neoplasm that causes multiple adenomatous polyps in the colon and is caused by a pathological variant of the adenomatous polyposis coli gene in the germline. The correlation between genotype and phenotype in FAP is useful for making decisions regarding its screening and surgical management [6]. With the advent of IPAA [7] and the development of the J-shaped ileal pouch [8], postoperative outcomes improved; however, a few patients experienced complications. Pouch-related complications (PRC) are one of the most important factors affecting the long-term outcomes, and some cases of PRC lead to permanent ileostomy [9, 10]. Among them, ileal pouch perforation is a rare, long-term complication of restorative proctocolectomy.

Several reports of ileal pouch perforation have been reported in the literature, all of which are listed below [11–20] (Table 1). Hsu and Leonid et al. reported perforation of the ileal pouch due to external factors, such as blunt trauma [11, 12]. In pregnant women with an enlarged uterus, increased pouch pressure associated with perforation in relation to adhesions were reported [13, 14]. Other perforations are caused by *Salmonella typhimurium* infection [15], volvulus and subsequent obstruction of the terminal ileum [16], and idiopathic spontaneous perforation of the pouch [17]. Shapiro et al. reported two cases associated with rapid ingestion of a high-fiber, high-calorie meal. In one patient, perforation occurred twice; however, the second perforation occurred 6 weeks postoperatively, which might have been an after-effect of the first pouch rupture [18]. Takahashi et al. reported that the combination of an enlarged J-pouch blind end and pouchitis could result in perforation [19]. Dogan et al. also deduced that perforation could be caused by pouchitis in the ileal J-pouch [20]. In our observed two perforations, stricture at the anastomosis was confirmed at both instances. Upon initial perforation, there was no ulcer on the mucosal surface in the resected specimen. Since endoscopy was not performed early following surgery, the existence of pouchitis could not be verified, despite dilatation of the pouch. Immediately before perforation, the patient also had symptoms of constipation. Therefore, the perforation was believed to be caused by increased intraluminal pressure in the pouch arising from an anastomotic stricture; this increased pressure was also suspected in the second perforation. Multiple ulcers in the pouch were confirmed by postoperative endoscopy; hence, pouchitis was also suspected to have contributed to the ileal pouch rupture. In addition, the results of the ascites culture test revealed only *E. coli*, which was believed to be derived from intestinal perforation and was not the cause of perforation.

**Table 1:** Reported cases of ileal pouch perforation

Author	Sex	Disease	Causes of perforation	Period
Lontoft [15]	F	UC	*Salmonella typhimurium* infection	2 y 6 m
Pezim [16]	M	UC	Volvulus of the terminal ileum	3 y 5 m
	M	UC	Volvulus of the terminal ileum	3 y 9 m
Hsu [11]	F	UC	Trauma	Unknown
Shapiro [18]	F	UC	Rapid consumption of high-fiber, high-calorie meals	2 y
	M	UC	Rapid consumption of high-fiber, high-calorie meals	1 y 8 m
Aouthmany [14]	F	UC	Pregnancy	10 y
Takahashi [19]	F	UC	The combination of an enlarged J-pouch blind end and pouchitis	8 y
Dogan [20]	M	FAP	Pouchitis	5 y
Panwar [17]	F	UC	Idiopathic	1 y
Drober [12]	M	UC	Trauma	20 y
Wasson [13]	F	UC	Pregnancy	Unknown
Our case	M	FAP	Intraluminal pressure in the pouch arising from anastomotic stricture and pouchitis	6 y and 18 y

*F*: female; *M*: male; *UC*: ulcerative colitis; *FAP*: Familial adenomatous polyposis; *Period*: the period from the primary surgery to the onset; *y*: years; m: months

Holubar et al. stated that obstruction from pouch–anal anastomosis stricture is common and requires surgical dilation with Hegar dilators and endoscopic balloon dilation. Needle–knife stricturotomy, chronic self-dilation at home, and a hand-sewn reanastomosis are required in refractory cases [21]. In our case, the anastomosis, which was dilated during the initial pouch perforation, was transiently maintained by self-bougie and constant observation. However, at some point, the self-bougie was not performed, and the patient’s follow-up was irregular. Accordingly, stenosis recurred, and pouchitis could not be primarily detected, which might have caused a second perforation. During that time, if the symptoms associated with the stricture, such as intestinal obstruction, were observed, early detection could have been possible. By doing so, his double perforation might have been prevented. Therefore, it is important to regularly observe such patients.

There are several reports on IPAA methods in patients with FAP. Konishi et al. reported that PRC in FAP patients were lower in stapled IPAA than in hand-sewn IPAA. In contrast, there were no differences in overall complication rates, fecal incontinence scores, ostomy rates, and overall survival between the two techniques. Therefore, they concluded that stapled IPAA might be a safer option for FAP patients to reduce PRC [22]. Ganschow et al. suggested that rectal mucosa, especially the wide mucosal seams and rectal adenomas, are often observed after a stapled than a hand-sewn anastomosis, which might be related to long-term outcomes [23]. Therefore, the preference between stapled IPAA or hand-sewn IPAA remains controversial. As Smith et al. stated [24], regardless of the anastomotic technique, careful regular surveillance and functional maintenance of pouches are critical.

## Conclusion

The blind end of the J-pouch repeatedly perforated over the years due to recurrent anastomotic stricture in our patient. Regular surveillance is necessary in these cases and may prevent perforation of the ileal pouch when strictures are released on time and anastomotic patency is maintained.

## Data Availability

The data are not available for public access because of patient privacy concerns but are available from the corresponding author on reasonable request.

## Abbreviations

APC: Adenomatous polyposis coli
CT: Computed tomography
CRP: C-reactive protein
FAP: Familial adenomatous polyposis
IPAA: Ileal pouch anal anastomosis
PRC: Pouch-related complication
RPC: Restorative proctocolectomy
WBC: White blood cell
